# Mental Health Support for the Current and Future Medical Professionals during Pandemics

**DOI:** 10.31662/jmaj.2021-0039

**Published:** 2021-07-09

**Authors:** Soichiro Saeki, Masako Shimato

**Affiliations:** 1 Faculty of Medicine, Medical School, Osaka University, Osaka, Japan; 2 Department of Global and Innovative Medicine, Graduate School of Medicine, Osaka University, Osaka, Japan; 3 UCL Medical School, Faculty of Medical Sciences, University College London, London, United Kingdom

**Keywords:** Medical Students, Burnouts, Clinical Clerkship, Medical Staff, COVID-19

## Abstract

Psychological distress among medical professionals due to the novel coronavirus disease 2019 (COVID-19) pandemic is of great concern as it may lead to mental health problems and, furthermore, work leaves. Studies suggest that immediate psychological interventions are needed to protect medical staff during this chaotic situation.

However, the importance of mental healthcare for the “future” medical staff, such as medical students, remains underestimated compared with that of current medical professionals. Medical students also face potential mental health stressors during their degrees and clinical clerkships, further increasing the risk of their psychological disorders. Hence, they should also be protected from burnout by various measures at governmental, organizational, and individual levels.

To maintain a sustainable, robust healthcare system, medical professionals, students, and society should unite and collaboratively overcome the hardships of the COVID-19 pandemic.

The novel coronavirus disease 2019 (COVID-19), now resulting in a global pandemic, has been casting severe burden on medical professionals. The physical and psychological stress of combating public health crises, such as the COVID-19 pandemic, could lead to mental health problems ^(1)^ and, furthermore, work leaves. Therefore, the mental status of medical professionals should be of concern both in short and long terms. In Japan, a vast number of medical staff are dropping out from work because of COVID-19-related issues ^(2)^. To the best of our knowledge, there are no official governmental statistics on medical staff sickness absence in Japan for 2020. However, studies from overseas ^(1), (3)^ suggest the need for immediate psychological interventions, regardless of the availability of statistics.

As an example, we hereby present the data of sickness absence in the National Health Service ^(4)^ in the United Kingdom (UK) (Figure 1). As COVID-19 cases surged in the UK in April 2020, the percentage of sickness absence due to psychological problems has been increasing since, which is responsible for nearly a third of all the sickness absence in July 2020, equivalent to over 483,000 full-time equivalent days lost. However, the total sickness absence did not remarkably increase in comparison to the sickness absence rate among medical staff from March to July in 2019 and 2020. While remaining steady around 4% in 2019, the rate was high in March, April, and May 2020. This implies that the psychological burden due to this pandemic becomes apparent subsequently to the outbreak surge. Therefore, mental health supports for medical professionals are needed during the toughest times to sustain the future healthcare system.

**Figure 1.**
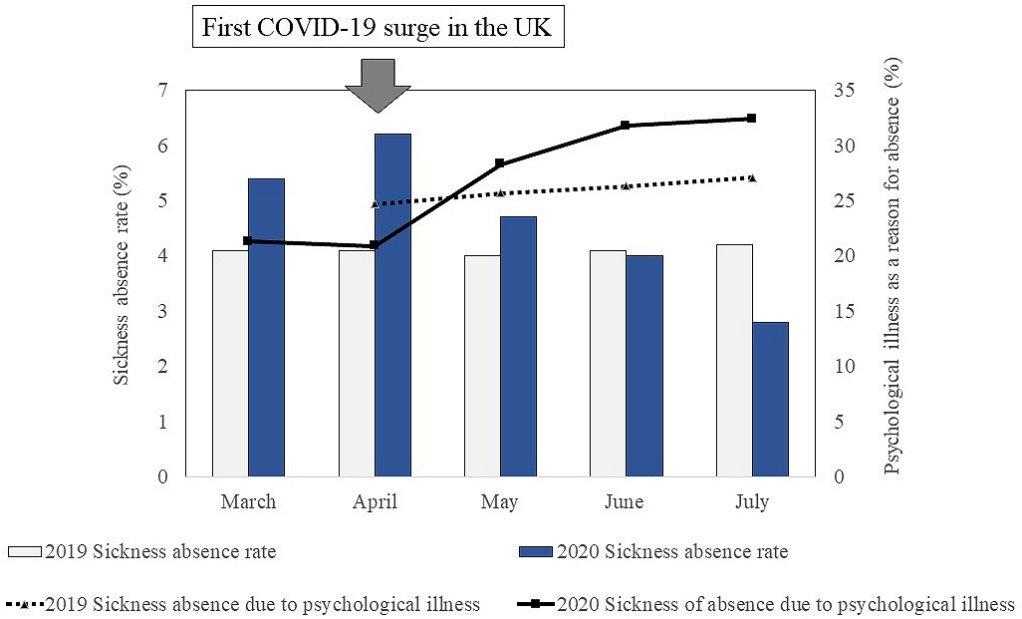
Sickness absence of National Health Service staff in 2019 and 2020. Data was analyzed using open-source data provided by the National Health Service ^(4)^.

This issue is not limited to Japan or the UK. A systematic review ^(5)^ of 59 studies worldwide shows that the prevalence of mental issues during the pandemic varies but is consistently high among the medical professionals: anxiety, 24%; depression, 21%; sleep problem, 37%; and distress, 37% (in median). Even in the population where psychological symptoms were not prevalent, physical symptoms such as headache were commonly reported ^(6)^.

Moreover, the focus of psychological care during a pandemic should not be limited to medical professionals. Timely, adequate mental care for medical students undertaking clinical clerkships needs further emphasis. Overseas studies suggest that medical students worldwide have been under psychological stress during the pandemic ^(3)^. Even without practical evidence of actual psychological burden on medical students in Japan, they should also be cared now to minimize the long-term impacts on their mental well-being ^(7)^.

There are several reasons why “future” physicians should be included in the psychological care focus during the pandemic. Exposure to patients with COVID-19 elevates the stress level even in medical students ^(3)^, potentially risking them for psychological illness along with other medical professionals. In addition, the social pressure against the infected students ^(8)^ cannot be dismissed. In Japan, the COVID-19 clusters of medical students were sensationally featured in the media. Students have been in fear of becoming the next target for this social punishment. Finally, of all the medical students, diligent, dedicated students have been the most stressed and frustrated with the reduced clinical exposure. These students may feel extensively insecure about their future careers as they get less clinical experience than those trained prior to the pandemic. Many medical schools worldwide had to amend, postpone, or cancel clinical training during the pandemic ^(9)^, which could lead to the lack of competency at the time of graduation.

To protect medical professionals from burnout due to the strenuous healthcare provision, some recommendations have been published since the beginning of the pandemic ^(1), (2)^. Modified to be applied to medical students, the following strategies should be considered. Before starting clinical clerkships, students should receive detailed inductions to explore and prepare for the anticipated challenges associated with potential exposure to patients with COVID-19 ^(1)^. To identify early signs of psychological distress and presenteeism at clinical placements, medical schools should show strong leadership and promote intimate communications with and among students, for example, by organizing regular reflection sessions, frequently collecting feedback from students about their clinical experiences during the pandemic to identify their needs, updating information on the measures taken by the medical schools in response to the pandemic, and introducing available mental health supports within the hospitals or medical schools ^(10)^. This will also help create a supportive and open atmosphere where medical students can safely discuss various experiences and emotions. Additionally, debrief and reflection sessions after the outbreak will help them interpret difficult episodes in a meaningful way ^(1)^.

To summarize, both current and future medical staff should be protected from burnout in this unprecedented situation by various measures at governmental, organizational, and individual levels. National governments should organize policies to ensure the mental well-being of all personnel in the medical field during pandemics. At the organizational level, the provision of adequate trainings and personal protective equipment as well as and the recognition of their efforts will improve both motivation and psychological outcomes among the medical professionals ^(11)^. Clear and frequent communication is the key in promptly identifying their pandemic-related needs, and up-to-date information should be provided to reduce their anxiety due to the uncertainty of the situation. To identify signs of psychological distress as early as possible, campaigns to reduce stigma against psychological issues in the workplace and peer support system can be introduced. The telephone-based hotline to psychiatrists or psychologist should be available to address individual issues and promptly offer adequate psychological supports. At the individual level, mindfulness interventions have been shown to be effective in reducing stress among healthcare staff by allowing them to objectify their emotions and negative thoughts ^(11)^. Talking to family members over telephones is considered by frontline workers as a very important coping mechanism. Mental support, especially for medical students, although often underestimated, should be highlighted. Protecting such students is of great importance in society, as the main purpose of medical education is to sustainably promote the health in each community.

Society members rely on healthcare staff for their physical and mental well-being. Conversely, medical professionals are also supported by society. Therefore, medical professionals, students, and society should unite to secure the mental safety of one another, so that we can collaboratively overcome the hardships of the COVID-19 pandemic and ensure a robust healthcare system in the future.

## Article Information

### Conflicts of Interest

None

### Acknowledgement

We express our deepest gratitude for the medical staff in combat against COVID-19. SS also thanks the Osaka University Medical Doctor Scientist Training Program and acknowledges Professors Manabu Ikeda and Toshihisa Tanaka for their helpful insights.

### Author Contributions

SS conceptualized the basic concept and made the synopsis of this manuscript. MS and SS analyzed the data, and MS created the key graphics. Both authors edited the first draft, read the final manuscript, and approved to the submission of this manuscript. Both authors equally contributed to this manuscript.

### Approval by Institutional Review Board (IRB)

Not applicable

## References

[1] Greenberg N, Docherty M, Gnanapragasam S, et al. Managing mental health challenges faced by healthcare workers during covid-19 pandemic. BMJ. 2020;368:m1211. 32217624 10.1136/bmj.m1211

[2] Matsuo T, Kobayashi D, Taki F, et al. Prevalence of health care worker burnout during the coronavirus disease 2019 (COVID-19) pandemic in Japan. JAMA Network Open. 2020;3(8):e2017271. 32749466 10.1001/jamanetworkopen.2020.17271 PMC7403916

[3] Wu S, Li Z, Li Z, et al. The mental state and risk factors of Chinese medical staff and medical students in early stages of the COVID-19 epidemic. Compr Psychiatry. 2020;102:152202. 32866693 10.1016/j.comppsych.2020.152202 PMC7437442

[4] NHS Sickness Absence Rates July 2020, Provisional Statistics [Internet]. c2020 [updated 2020 Nov; cited 2020 Mar 11]. Available from: https://digital.nhs.uk/data-and-information/publications/statistical/nhs-sickness-absence-rates/july-2020.

[5] Muller AE, Hafstad EV, Himmels JPW, et al. The mental health impact of the covid-19 pandemic on healthcare workers, and interventions to help them: a rapid systematic review. Psychiatry Res. 2020;293:113441. 32898840 10.1016/j.psychres.2020.113441 PMC7462563

[6] Ng QX, De Deyn M, Lim DY, et al. The wounded healer: a narrative review of the mental health effects of the COVID-19 pandemic on healthcare workers. Asian J Psychiatr. 2020;54:102258. 32603985 10.1016/j.ajp.2020.102258 PMC7305497

[7] Albott CS, Wozniak JR, McGlinch BP, et al. Battle buddies: rapid deployment of a psychological resilience intervention for health care workers during the COVID-19 pandemic. Anesth Analg. 2020;131(1):43-54. 32345861 10.1213/ANE.0000000000004912 PMC7199769

[8] Do Our Bit Student’s Project, Nishihara M, Ota Y, et al. Mandatory? or Voluntary? Student survey on the restraint due to the COVID-19 pandemic. Journal of International Health. 2020;35(2):93-5. Japanese.

[9] Yamasaki L, Saeki S, Kido H, et al. Medical students in the midst of COVID-19 -Report of the joint student symposium in the Joint Congress of Global Health 2020 in Osaka-. Journal of International Health. 2020;35(4):265-6.

[10] Cai W, Lian B, Song X, et al. A cross-sectional study on mental health among health care workers during the outbreak of Corona Virus Disease 2019. Asian J Psychiatr. 2020;51:102111. 32361388 10.1016/j.ajp.2020.102111 PMC7194661

[11] Galbraith N, Boyda D, McFeeters D, et al. The mental health of doctors during the COVID-19 pandemic. BJPsych Bull. 2021;45(2):93-7. 32340645 10.1192/bjb.2020.44 PMC7322151

